# Effects of Exogenous Abscisic Acid on Bioactive Components and Antioxidant Capacity of Postharvest Tomato during Ripening

**DOI:** 10.3390/molecules25061346

**Published:** 2020-03-16

**Authors:** Xiaoya Tao, Qiong Wu, Halah Aalim, Li Li, Linchun Mao, Zisheng Luo, Tiejin Ying

**Affiliations:** 1 College of Biosystems Engineering and Food Science, Fuli Institute of Food Science, Zhejiang Key Laboratory for Agro-Food Processing, Zhejiang R & D Center for Food Technology and Equipment, Zhejiang University, Hangzhou 310058, China; taoxiaoya00@163.com (X.T.);; 2 Collaborative Innovation Center of Henan Grain Crops, Henan Collaborative Innovation Center of Grain Storage and Security, School of Food Science and Technology, Henan University of Technology, Zhengzhou 450001, China

**Keywords:** tomato, abscisic acid, bioactive components, enzymatic activity, antioxidant capacity, gene expression

## Abstract

Abscisic acid (ABA) is a phytohormone which is involved in the regulation of tomato ripening. In this research, the effects of exogenous ABA on the bioactive components and antioxidant capacity of the tomato during postharvest ripening were evaluated. Mature green cherry tomatoes were infiltrated with either ABA (1.0 mM) or deionized water (control) and stored in the dark for 15 days at 20 °C with 90% relative humidity. Fruit colour, firmness, total phenolic and flavonoid contents, phenolic compounds, lycopene, ascorbic acid, enzymatic activities, and antioxidant capacity, as well as the expression of major genes related to phenolic compounds, were periodically monitored. The results revealed that exogenous ABA accelerated the accumulations of total phenolic and flavonoid contents; mostly increased the contents of detected phenolic compounds; enhanced FRAP and DPPH activity; and promoted the activities of PAL, POD, PPO, CAT, and APX during tomato ripening. Meanwhile, the expressions of the major genes (*PAL1*, *C4H*, *4CL2*, *CHS2*, *F3H*, and *FLS*) involved in the phenylpropanoid pathway were up-regulated (1.13- to 26.95-fold) in the tomato during the first seven days after treatment. These findings indicated that ABA promoted the accumulation of bioactive components and the antioxidant capacity via the regulation of gene expression during tomato ripening.

## 1. Introduction

The tomato (*Solanum lycopersicum*) is a popular fruit which is consumed worldwide [1]. The nutritional and functional attributes of the tomato are mainly estimated by the accumulation of bioactive components, such as ascorbic acid, phenolics, and flavonoids [2,3], as well as the antioxidant activity. The consumption of tomatoes is associated with the reduced risk of cancer and cardiovascular diseases [4] due to the antioxidant and anti-inflammatory activities of phenylpropanoid metabolites [5,6,7].

Phenolics are major secondary metabolites in fruits and vegetables which have potent antioxidative activity [8]. The phenolic contents of the tomato are influenced by the ripening stage, and there are more of them in green and medium-ripened tomatoes than in fully ripened tomatoes [9,10]. Studies have shown that tomato-derived ascorbic acid and phenolic compounds are associated with numerous health-promoting properties [11,12]. Furthermore, lycopene is a potent natural antioxidant owing to its excellent quenching ability [13,14]; it also has anti-carcinogenic and anti-atherogenic potentials [15].

Abscisic acid (ABA) is a plant growth regulator which is involved in fruit ripening by its own or by interacting with other plant hormones [16,17,18,19]. Furthermore, ABA can affect the synthesis of metabolites in plants. Exogenous ABA could increase the phenolic contents in muscadine grapes [20] and maize root [21], in addition to the antioxidant activity in kiwifruit plants [22], maize leaves [23], grapevine leaves [24], and maize root [21]. In addition, exogenous ABA activates phenylpropanoid and flavonol biosynthesis and enhances the gene expression of grape skins [25,26]. In the strawberry fruit, there was a positive correlation between ABA and phenolic compounds, and ABA and the corresponding gene expression [27].

To date, there is a lack of knowledge about the effects of exogenous ABA on the bioactive components and the gene expression of the phenylpropanoid pathway during tomato ripening. Therefore, the current study evaluated the changes in antioxidant activity and the related bioactive components, enzymatic activities, and the corresponding gene expression after exogenous-ABA application during tomato ripening.

## 2. Results

### 2.1. Effect of ABA on Colour and Firmness

**Figure 1:**
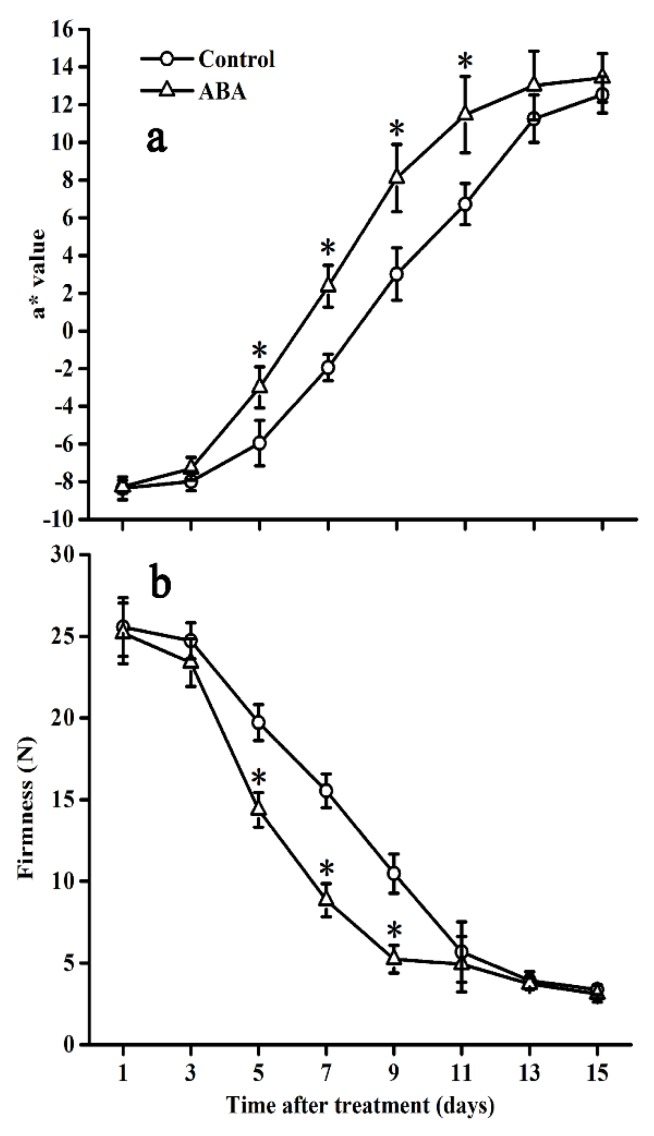
Effect of abscisic acid (ABA) on colour (**a**) and firmness (**b**) in the tomato during storage at 20 °C. Vertical bars represent the standard deviation (SD, n = 3). Asterisks (*) represent significant differences (*p* < 0.05) between the ABA and control treatments.

As shown in Figure 1, fruit colour showed an upward trend, whereas fruit firmness displayed an opposite trend in both control and ABA-treated fruits. Significant acceleration in both fruit colour and firmness was observed between ABA and control fruit from day 5 to day 9. However, no significant differences were observed between the two groups at the end of the storage period.

### 2.2. Effect of ABA on Total Phenolic and Flavonoid Contents

**Figure 2:**
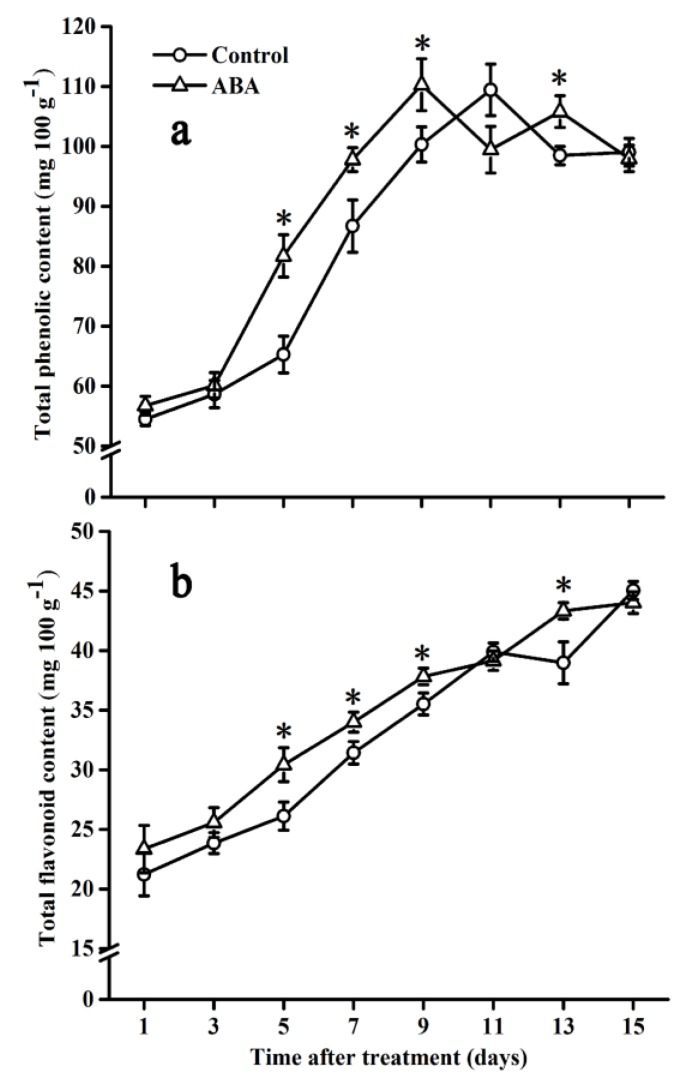
Effect of ABA on the total phenolic (**a**) and flavonoid (**b**) content in the tomato during storage at 20 °C. Vertical bars represent the standard deviation (SD, n = 3). Asterisks (*) represent significant differences (*p* < 0.05) between the ABA and control treatments.

The changes in total phenolic and flavonoid contents are shown in Figure 2. Total phenolic content in control fruits increased rapidly during the first 11 days and reached the maximum, followed by a decrease, at the end of the storage time (Figure 2a). A similar pattern was observed in ABA-treated fruits: total phenolic content was significantly higher during the first nine days when compared with the control. In addition, the peak in the ABA group was two days earlier than that in the control group. Total flavonoid content increased gradually in both groups during the storage period (Figure 2b). Specifically, in comparison with control fruits, ABA treatment accelerated the increase in total flavonoid content in the tomato from day 5 to day 13, except for day 11.

### 2.3. Effect of ABA on Phenolic Compounds

**Figure 3:**
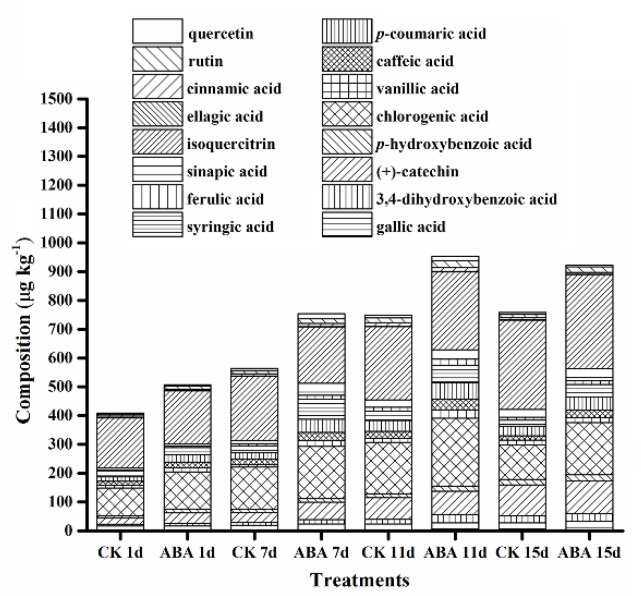
The visual results of the composition of phenolic compounds in the tomato at the mature green, breaker, turning, and red stages, respectively.

To explore the effect of exogenous ABA on phenolic compounds during tomato ripening, the contents of phenolic compounds were analyzed with the chosen samples on days 1, 7, 11, and 15, which represented the mature green, breaker, turning, and red stages, respectively. The results are shown in Figure 3. A total of 16 phenolic compounds were detected in the current research.

In general, the total content of phenolic compounds in both groups increased gradually during tomato ripening, as exogenous ABA could promote the accumulation of almost all of the detected phenolic compounds besides the total phenolic contents. Specifically, isoquercitrin, chlorogenic acid, (+)-catechin, *p*-coumaric acid, and syringic acid were the major phenolic compounds. The content of isoquercitrin and (+)-catechin in both groups increased gradually during ripening. The content of chlorogenic acid and *p*-coumaric acid, however, showed a tendency to increase first and reach the maximum on day 11, followed by significant reduction. The contents of the rest of the phenolic compounds were relatively low and declined eventually.

### 2.4. Effect of ABA on Lycopene and Ascorbic Acid Contents

**Figure 4:**
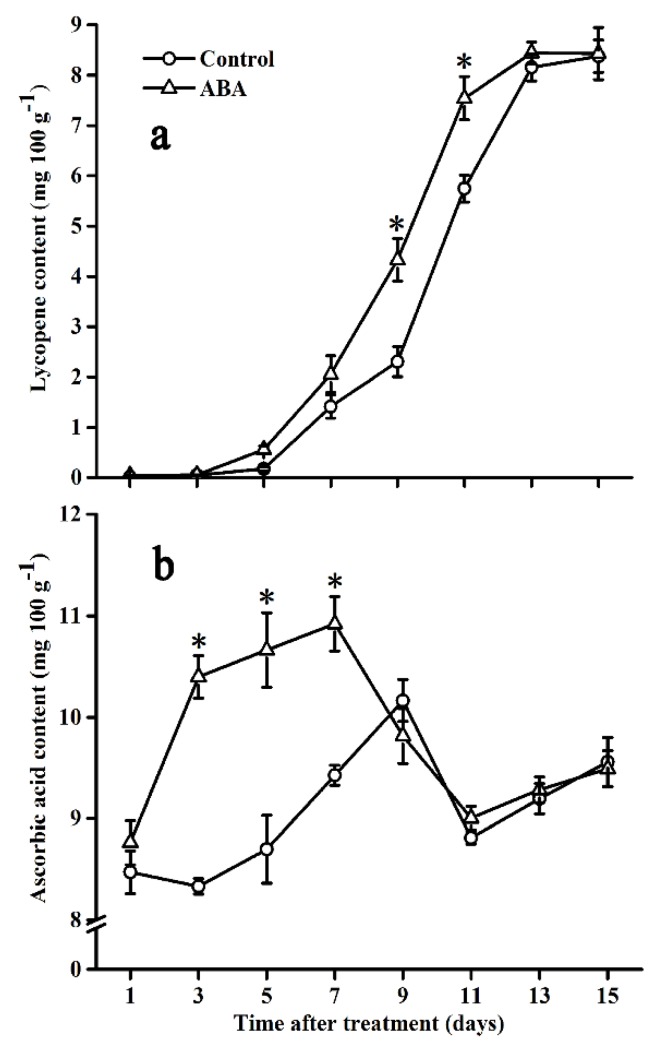
Effect of ABA on lycopene (**a**) and ascorbic acid (**b**) contents in the tomato during storage at 20 °C. Vertical bars represent the standard deviation (SD, n = 3). Asterisks (*) represent significant differences (*p* < 0.05) between the ABA and control treatments.

Lycopene content in both groups was undetectable until day 5, and then a rapid increase was observed during the subsequent storage period (Figure 4a). A significant increase in the lycopene content in ABA-treated fruits was shown on day 9 and day 11, which was 1.45- and 1.10-fold, respectively, compared with that in control fruits.

As shown in Figure 4b, the ascorbic acid content in control fruits increased gradually before reaching the maximum on day 9, followed by a rapid decrease and a slight increase during the end of the ripening stage. Compared with control fruits, the ascorbic acid content in ABA-treated fruits increased significantly from day 3 to day 7, and the peak value was two days earlier. However, no significant differences were observed after seven days.

### 2.5. Effect of ABA on PAL, POD, and PPO Activities

**Figure 5:**
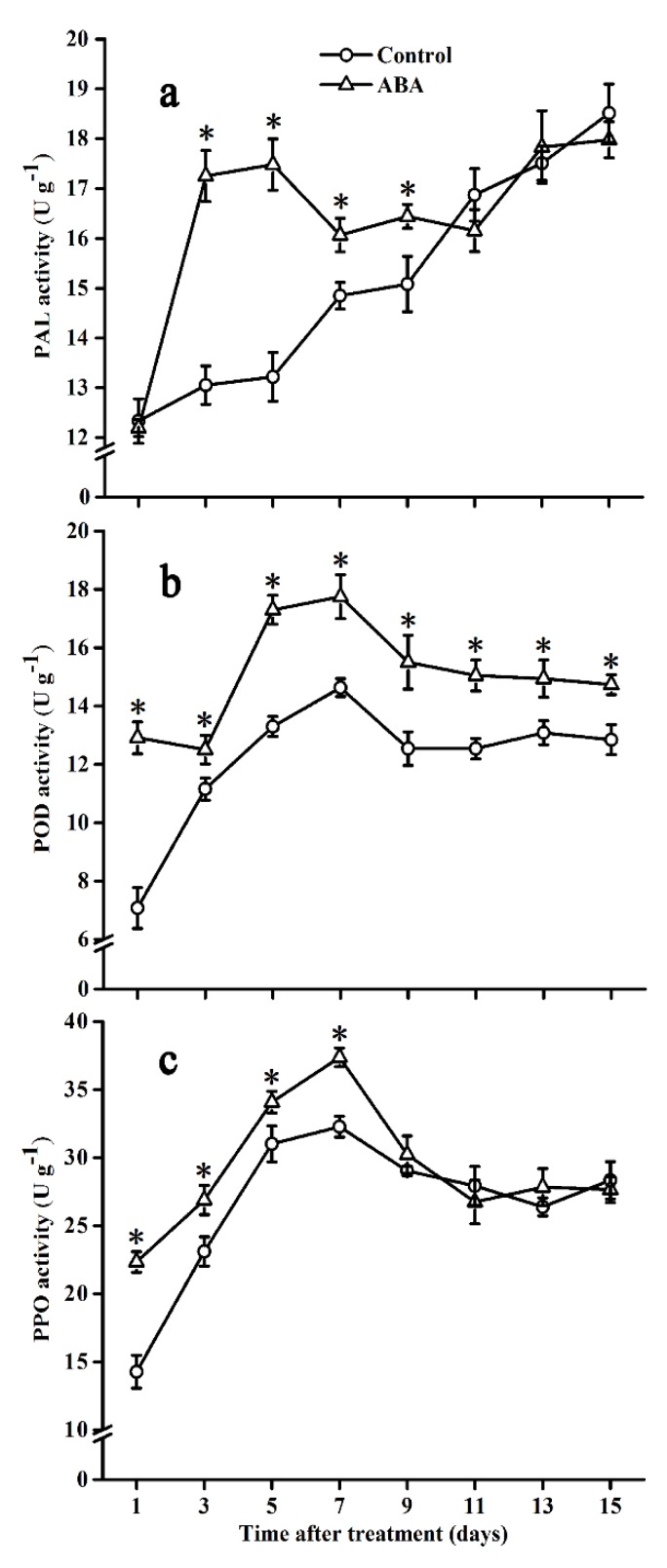
Effect of ABA on PAL (**a**), POD (**b**), and PPO (**c**) activities in the tomato during storage at 20 °C. Vertical bars represent the standard deviation (SD, n = 3). Asterisks (*) represent significant differences (*p* < 0.05) between the ABA and control treatments.

PAL activity in control fruits gradually increased over the storage period (Figure 5a), whereas a significant increase due to ABA application was shown from day 3 to day 9 compared with the control. As shown in Figure 5b, POD activity in both control and ABA-treated fruits gradually increased up to day 7, followed by a slight decrease on day 9, and then kept a relatively stable level until the end of the storage time. POD activity significantly increased after ABA treatment during the entire storage period. PPO activity in both groups exhibited a similar trend as POD (Figure 5c). Exogenous ABA significantly promoted PPO activity from day 1 to day 7.

### 2.6. Effect of ABA on CAT and APX Activities

**Figure 6:**
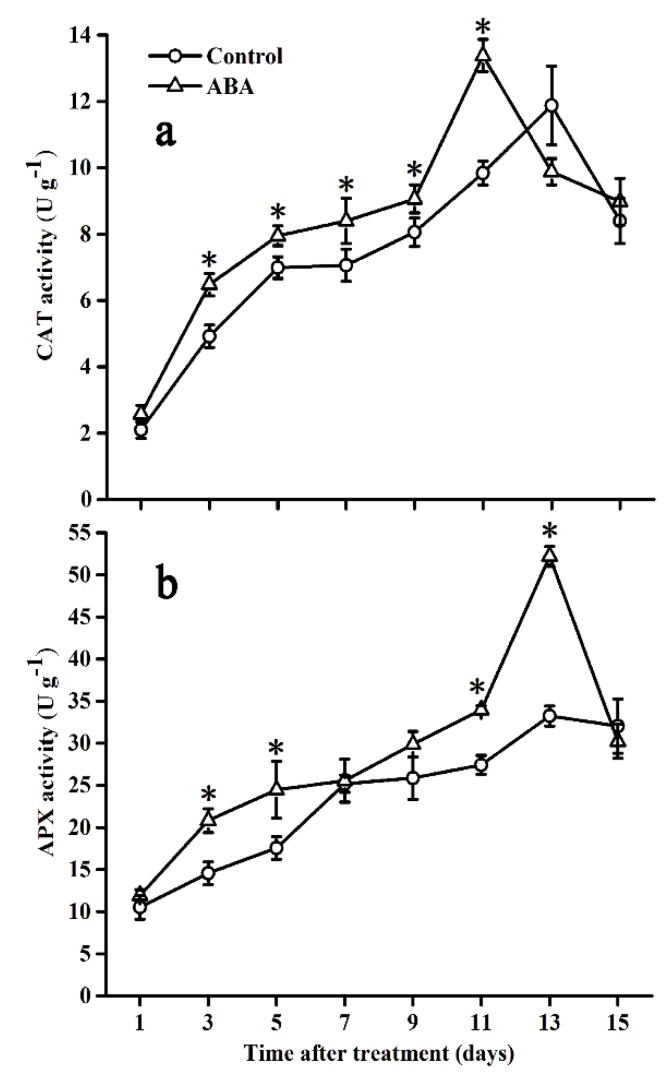
Effect of ABA on CAT (**a**) and APX (**b**) activities in the tomato during storage at 20 °C. Vertical bars represent the standard deviation (SD, n = 3). Asterisks (*) represent significant differences (*p* < 0.05) between the ABA and control treatments.

CAT and APX activities showed similar behaviors in both groups during tomato ripening (Figure 6). In control fruits, both activities increased rapidly up to 13 days, and then decreased sharply or slightly at the end of the storage period, respectively. Compared with control fruits, in ABA-treated fruits, both of them increased rapidly and reached a higher level on day 11 and day 13, respectively, followed by a sharp decline at the rest of the storage time.

### 2.7. Effect of ABA on Antioxidant Capacity

**Figure 7:**
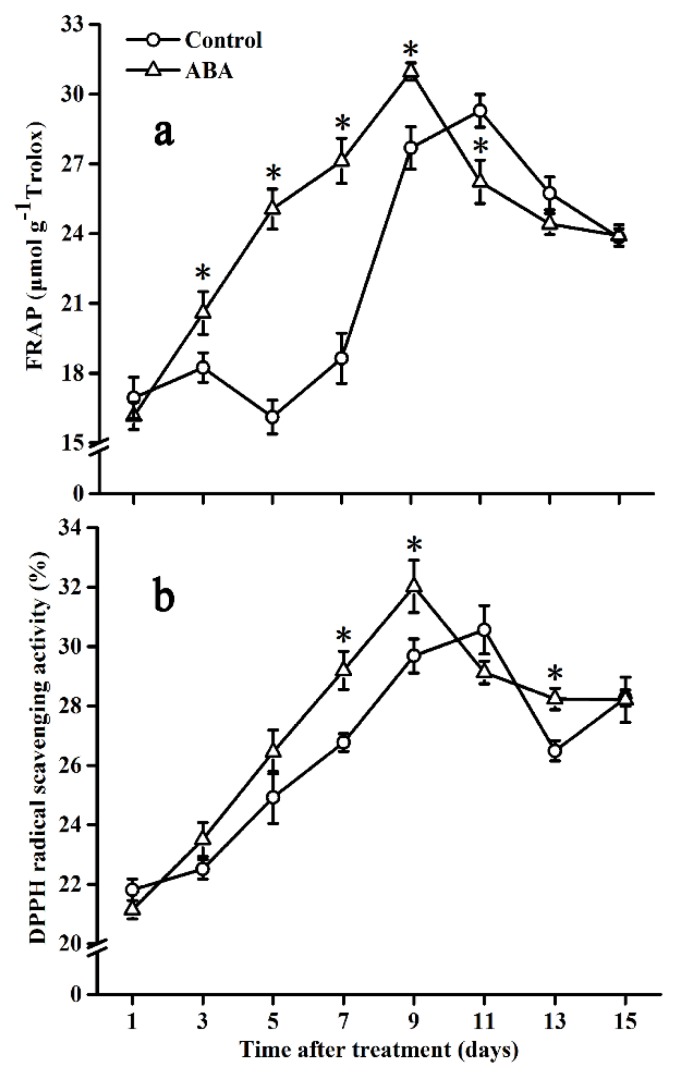
Effect of ABA on total antioxidant capacity FRAP (**a**) and DPPH radical scavenging activity (**b**) in the tomato during storage at 20 °C. Vertical bars represent the standard deviation (SD, n = 3). Asterisks (*) represent significant differences (*p* < 0.05) between the ABA and control treatments.

The antioxidant capacity was evaluated by FRAP and DPPH radical scavenging activity (Figure 7). In control fruits, FRAP was relatively stable before day 7, followed by a rapid increase and reaching the maximum value on day 11, then decreasing gradually (Figure 7a). FRAP in ABA-treated fruits showed a sharp increase until day 9, then decreased rapidly with no differences at the end of the storage time, except for day 11, in which FRAP was lower than that in control fruits.

DPPH radical scavenging activity showed a similar pattern with FRAP in both groups during the storage time (Figure 7b). In addition, DPPH radical scavenging activity was significantly higher in ABA-treated fruits on day 7, day 9, and day 13 when compared with that in control fruits.

### 2.8. Effect of ABA on Gene Expression

**Figure 8:**
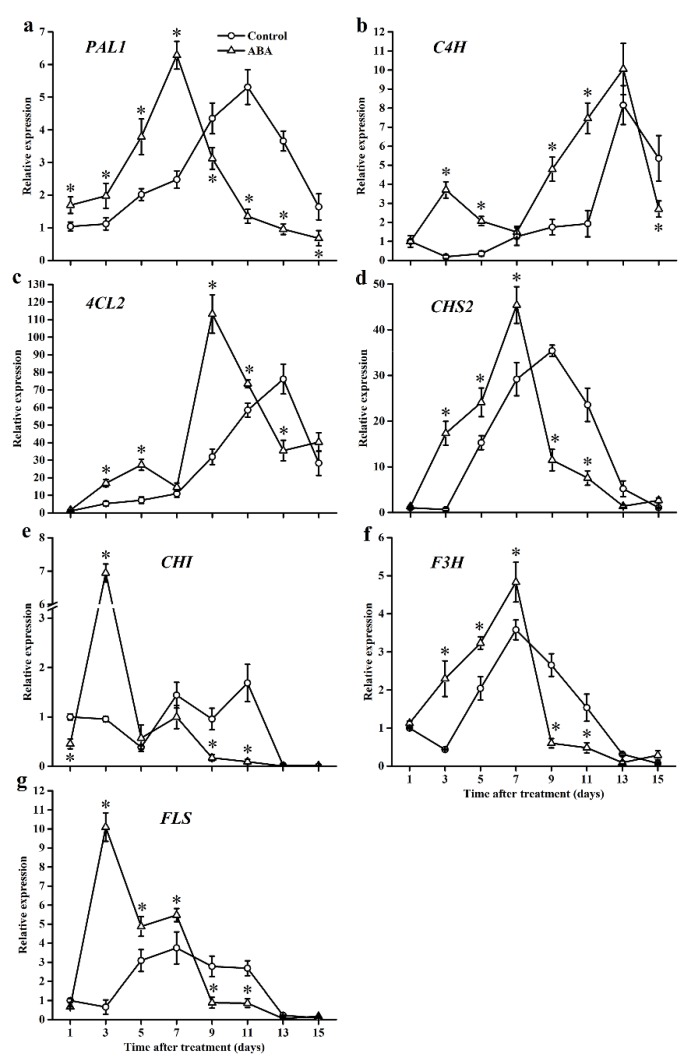
Effect of ABA on the gene expression of the phenylpropanoid pathway in the tomato during storage at 20 °C. Vertical bars represent the standard deviation (SD, n = 3). Asterisks (*) represent significant differences (*p* < 0.05) between the ABA and control treatments.

Seven major genes were selected to evaluate the effect of exogenous ABA on the phenylpropanoid pathway, and the results are shown in Figure 8. Specifically, *PAL1* and *CHS2* showed similar patterns, and their expressions in control fruits increased and reached the maximum value on day 11 and day 9, respectively. Exogenous ABA resulted in higher and earlier peaks on day 7. Compared with control fruits, exogenous ABA significantly induced the expression of *C4H* and *4CL2*, whereas it inhibited them at the end of the storage time. The expression of *CHI* in control fruits kept a low level during the storage time and was significantly increased by exogenous ABA on day 3 (7.26-fold). The expression of *F3H* and *FLS* increased at the early stage of the storage period and then declined in both groups. Exogenous ABA significantly increased the expression of *F3H* and *FLS* during the first seven days, then significantly decreased on day 9 and day 11.

### 2.9. Correlation Between Gene Expression, Phenolic Compounds, and Antioxidant Capacities

**Figure 9:**
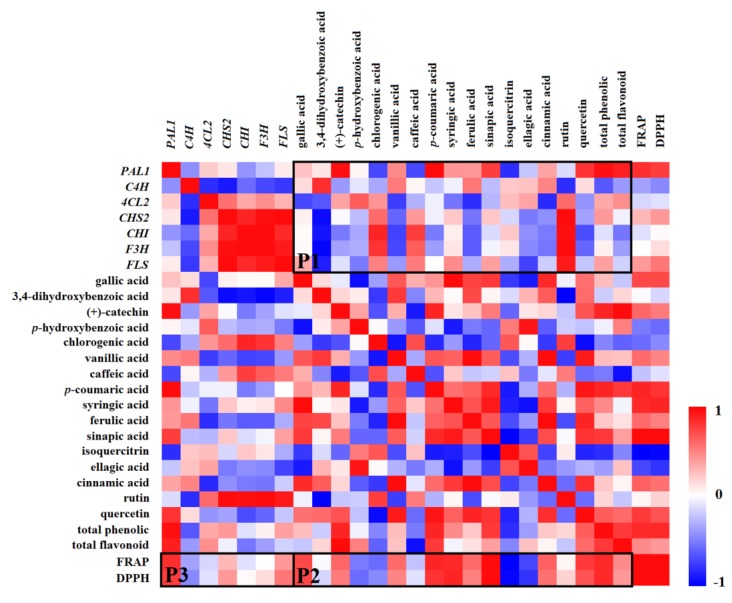
Effect of ABA on correlations between gene expression, phenolic compounds, and antioxidant capacities. The heatmap was produced based on obtained Pearson’s correlation coefficients with the relative levels of all indexes in ABA-treated fruits (the levels in control fruits were all normalized to one) on days 1, 7, 11, and 15. The red (+1) and blue (−1) colours represent the positive and negative correlations, respectively, between different indexes. (For interpretation of the references to colour in the Figure 9 legend, the reader is referred to the web version of this article.)

To study the effect of exogenous ABA on phenolic compounds during tomato ripening, correlation analysis between gene expression, phenolic compounds, and antioxidant capacities in ABA-treated fruits was analyzed and a heatmap was produced based on the obtained Pearson’s correlation coefficients (r) (Figure 9). The areas P1, P2, and P3 represent the correlations between the relative values (RVs) of genes and phenolic compounds, phenolic compounds and antioxidant capacities, and genes and antioxidant capacities, respectively.

As shown in Figure 9, in the P1 area, the correlations between genes and phenolic compounds were complex and varied. In general, rutin was positively correlated with all genes, except for *PAL1* and *C4H*. (+)-catechin (r^2^ = 0.95), *p*-coumaric acid (r^2^ = 0.97), and total phenolic (r^2^ = 0.95) were significantly positively correlated with *PAL1*. The correlation coefficient was high between quercetin (r^2^ = 0.80), total flavonoid (r^2^ = 0.89), and *PAL1*; *CHI* (r^2^ = 0.87), *F3H* (r^2^ = 0.81), and chlorogenic acid. However, ferulic and cinnamic acids were negatively correlated with most of the genes.

In the P2 area, most (12/18) of the phenolic compounds were positively correlated with antioxidant capacities. Specifically, significantly positive correlations between sinapic acid and FRAP (r^2^ = 0.99), and sinapic acid and DPPH (r^2^ = 0.98) were observed. However, negative correlations between isoquercitrin and FRAP (r^2^ = −0.99), and isoquercitrin and DPPH (r^2^ = −0.96) were shown. In addition, gallic acid, *p*-coumaric acid, syringic acid, quercetin, and total phenolic content were positively correlated with FRAP and DPPH, but they were negatively correlated with the chlorogenic and caffeic acids.

In the P3 area, in general, most of the genes were positively correlated with antioxidant capacities, except for *C4H* and *4CL2*. The correlation coefficients between the antioxidant capacities (FRAP and DPPH) and the genes *PAL1* (r^2^ = 0.83 and 0.77) and *FLS* (r^2^ = 0.42 and 0.54) were high. However, correlation coefficients were relatively low between antioxidant capacities and the other three genes.

## 3. Discussion

Colour and firmness are the vital features of the global quality of food products and are closely associated with fruit ripening [28]. In the present research, exogenous ABA treatment was found to promote tomato ripening and accelerate changes in fruit colour and firmness, as well as the accumulation of lycopene. This was consistent with the reported results that ABA accelerates the ripening process of the tomato [18,29,30,31]. In addition, total phenolic and flavonoid contents, phenolic compounds, enzymatic activities, and antioxidant capacities were generally enhanced after ABA application. These findings might be explained by the ABA treatment that seems to induce the phenolic accumulation, owing to the activation of the genes involved in the phenylpropanoid pathway.

**Figure 10:**
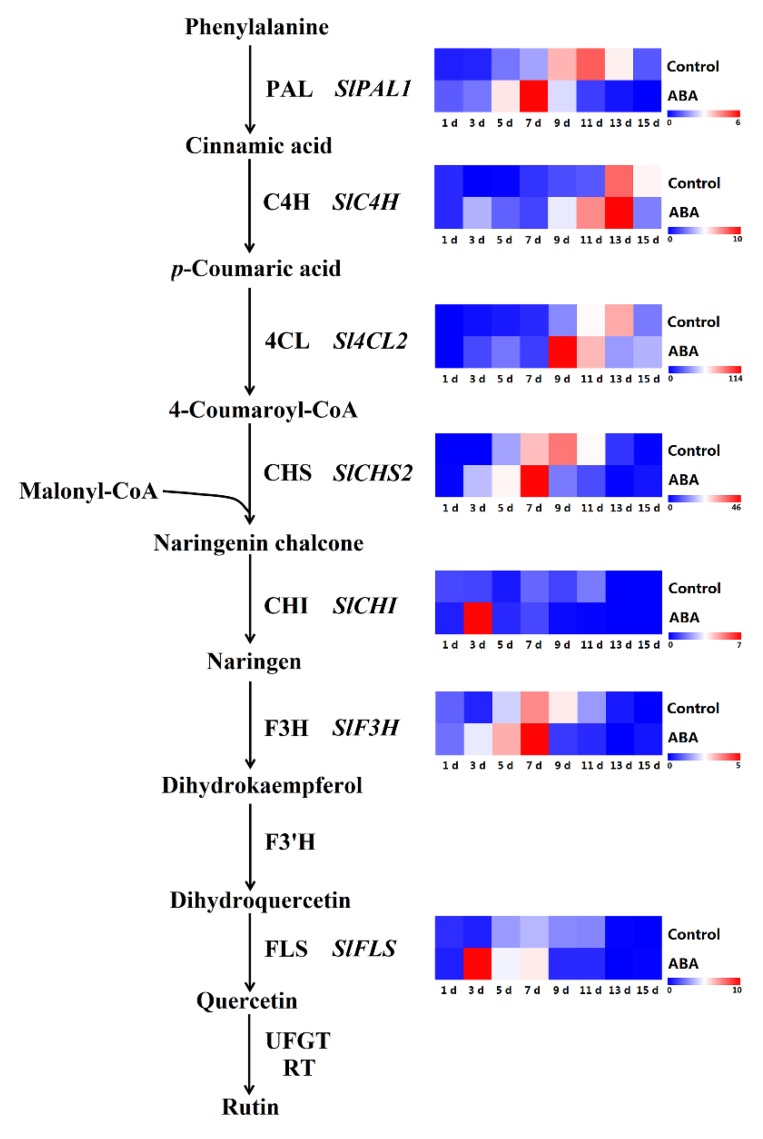
Schematic diagram of the phenylpropanoid pathway and the expression of partial genes involved in this pathway. The control treatment was used as a reference sample for gene expression analysis, calculated based on the 2^−ΔΔCt^ method. The results are shown in the heatmap. The red and blue colours represent the maximum and minimum expression levels, respectively. Enzyme names are abbreviated as follows: PAL, phenylalanine ammonia-lyase; C4H, cinnamate 4-hydroxylase; 4CL, 4-coumarate-CoA ligase; CHS, chalcone synthase; CHI, chalcone isomerase; F3H, flavanone 3-hydroxylase; FLS, flavonol synthase; UFGT, UDP flavonoid glucosyltransferase; RT, flavonoid 3-*O*-glucoside-rhamnosyltransferase.

Phenolic compounds, a group of bioactive molecules distributed in many plant species, can be affected by environmental stresses and exogenous elicitors [32]. In addition, flavonoids were reported to be the major phenolics in the tomato [33]. Phenolics and flavonoids are major plant antioxidants that could mitigate oxidative stress and protect the cellular structure from damage [34,35]. In the present research, we identified isoquercitrin, chlorogenic acid, (+)-catechin, *p*-coumaric acid, syringic acid, caffeic acid, and gallic acid as the major phenolic compounds in the tomato, which was in accordance with the results reported previously [36,37,38]. The major phenolic compounds of the tomato vary due to the differences in cultivars, cultivation conditions, harvesting time, and processing conditions [39,40]. Reports suggested that exogenous ABA enhanced the secondary metabolism of grapes [20]. The present study indicated that the contents of total phenolics and flavonoids were remarkably increased in ABA-treated fruits compared with that in control fruits, which was consistent with the results reported in the ABA-treated Chinese cabbage [41], Chinese kale [42], lettuce [43], and broccoli sprouts [32]. Exogenous ABA might intensify the response of the plant to developmental and environmental signals, and might promote the biosynthesis of phenolic compounds in fruits and other plant organs [20]. In the present study, antioxidant capacity (FRAP and DPPH) increased significantly in the ABA-treated tomato compared with that of the control during ripening, and the trends coincided with that of total phenolics, which indicated that they were the major antioxidant contributors in the tomato. Thus, the results indicate that ABA may promote antioxidant capacity by enhancing the accumulation of total phenolics during tomato ripening. In fact, the positive relationship between total phenolics and total flavonoids and FRAP, DPPH, and *PAL1* was demonstrated by correlation analysis in the present research, indicating that the increase in total phenolics and flavonoids is due, at least in part, to the up-regulation of *PAL1* expression. Meanwhile, our results indicated that almost all cases of major-gene expression (*PAL1*, *C4H*, *4CL2*, *CHS2*, *F3H,* and *FLS*), except for *CHI,* were up-regulated, and the activities of major enzymes (PAL, PPO, and POD) were improved by the exogenous-ABA treatment during the early storage period (Figure 10), which was in accordance with the studies of the grape berry [26,44] and the strawberry fruit [45], suggesting that the transcriptional regulation could be an important mechanism of the ABA in the regulation of phenolic compound biosynthesis.

Ascorbic acid, a major antioxidant, is known to protect plants from oxidative stress and have health-promoting qualities for the consumers [46,47]. In the present research, the ascorbic acid content in the tomato rapidly increased during the first seven days after the ABA treatment, which was similar to that in the grape [48] and Arabidopsis thaliana [49], as the ascorbic acid content was improved by the ABA treatment through promoting its recycling. The results indicated that ABA has a positive effect on promoting the accumulation of ascorbic acid in the tomato during the early ripening stage. Afterwards, ascorbic acid content decreased in ABA-treated fruits, which was two days earlier than that in control fruits. This phenomenon may be owing to the detoxification of H_2_O_2_ by the APX-catalyzed peroxidation of ascorbic acid which generates MDHA [50]. In general, H_2_O_2_ content increased during the early ripening stage and reached the maximum during the middle ripening stage of the tomato (unpublished data). To mitigate oxidative stress and protect the cellular structure from damage, the conversion of H_2_O_2_ into H_2_O was catalyzed, and ascorbic acid was catalyzed by APX to generate MDHA. Thus the ascorbic acid content decreased in both control and ABA-treated fruits. These findings suggest that ABA can enhance the detoxification process of H_2_O_2_ during the middle ripening stage of the tomato. In this research, we found that exogenous ABA significantly promoted the activities of CAT and APX during tomato ripening. The finding was consistent with the results of kiwifruit plants [22], maize seedlings [23,51,52], apple rootstocks [53], and turnip plants [54], which had been treated by exogenous ABA. In addition, exogenous ABA also elevated CAT activity in wheat [55], maize [56], rice leaves [57], and rice grains [58]. The results may be explained by the fact that ABA can result in the accumulation of H_2_O_2_, serving as a signalling molecule for the activation of antioxidant enzymes [56].

## 4. Materials and Methods

### 4.1. Plant Materials

Cherry tomatoes (*Solanum lycopersicum,* L. cv. “Xin Taiyang”) were manually picked at mature green stage based on the uniform shape and size, with no injuries and infections, from a standardized greenhouse (Transfar Agriculture Co. Ltd., Xiaoshan, Zhejiang, China), which provided standard culture temperature (20–25 °C) and relative humidity (RH, 70–85%). Tomatoes with three biological replicates used in the current research were harvested on May 13, 2019, June 3, 2019, and June 21, 2019. The fruits were immediately transported to the laboratory within two hours. The fruit surface was sanitized with 0.3% (*v*/*v*) sodium hypochlorite for 3 min, washed thrice with distilled water, and air-dried. A total of 700 fruits were selected and randomly divided into two groups after removing pedicels. The entire experiment was conducted in triplicate.

### 4.2. ExogenousABA Treatment

Fruits of the two groups were infiltrated with either 1.0 mM ABA (98%, Aladdin Industrial Inc., Shanghai, China) or deionized water (control) in a vacuum (0.06 MPa, 3 min), respectively. Afterwards, all fruits were air-dried and stored in darkness for 15 days (20 °C, 90% RH). Samples of pericarps from 8 fruits were taken randomly every 2 days, immediately frozen in liquid nitrogen, and kept at −80 °C for subsequent analysis. The concentration and treatment conditions of ABA were determined according to the preliminary experiments (data not shown).

### 4.3. Determination of Fruit Colour and Firmness

Twelve fruit with three replicates for each treatment were used to determine fruit colour at each time point. Four symmetrical positions around the equator on each fruit were measured with a Chroma Meter (Konica Minolta, CR-400, Hino-shi Tokyo, Japan), and the result was represented as a* value.

The twelve fruit mentioned above were then used to measure fruit firmness. Fruit firmness was measured using a texture analyzer (TA-XT2i, Stable Microsystems Texture Technologies Inc., Tokyo, UK), equipped with a 5-mm diameter circular probe, by penetrating the fruit by 12 mm at a speed of 1 mm s^−1^ on four symmetrical positions around the equator of the fruit without fruit skin. The result was expressed using the maximum force in Newtons (N).

### 4.4. Analysis of Total Phenolic and Flavonoid Contents

The frozen tomato fruit samples were ground into fine powder using liquid nitrogen. Total phenolic and flavonoid contents were extracted and assayed according to the method depicted by Toor and Savage [59] with some modifications. The frozen powder sample (about 1 g) was homogenized in 6 mL of cold ethanol (40%, *v/v*). After being extracted in a water bath at 60 °C for 1 h, the homogenate was centrifuged at 4 °C with 9000× *g* for 15 min. The supernatant was used for the analysis of total phenolic and flavonoid contents. Total phenolic content was measured with the Folin–Ciocalteu reagent and 7.5% (*m/v*) Na_2_CO_3_ and expressed as gallic acid equivalents based on fresh weight, in mg 100 g^−1^. Total flavonoid content was determined with a NaNO_2_-Al(NO_3_)_3_-NaOH reagent and expressed as rutin equivalents based on fresh weight, in mg 100 g^−1^.

### 4.5. Analysis of Phenolic Compounds

The extraction of phenolic compounds was performed as described by Vallverdu-Queralt et al. [60] with modifications. Briefly, about 1.0 g of fine powder sample was homogenized with 5 mL of 80% cold methanol, and the mixture was extracted overnight at 4 °C. After being centrifuged, the supernatant was dried under nitrogen flow with a digital dry bath (ND100-1, Ruicheng Instrument Co. Ltd., Hangzhou City, China). Lastly, the residue was dissolved in deionized water up to 2 mL and then filtrated through a 0.45-μm filter prior to high-performance liquid chromatography (HPLC).

HPLC analysis was performed on a Waters e2695 system (Waters Corporation, Milford, MA, USA) with an Agilent Zorbax SB-C18 column (5 μm, 250 × 4.6 mm, Agilent Technologies Co. Ltd., Santa Clara, CA, USA); the column temperature was 40 °C. The absorbance was detected ranging from 240 to 450 nm. Solvent A (phosphoric acid solution, *v/v*, 0.5%) and B (methanol for HPLC, 100%) were used with a gradient profile including A with the following proportions (*v/v*) of B: 0–5 min, 10–15% B; 5–45 min, 15–28.3% B; 45–72 min, 28.3–71.7% B; 72–75 min, 71.7–10% B; and 75–78 min, 10–10% B. The flow rate was 1.0 mL min^−1^ and the injection volume was 20 μL. Phenolic compounds were identified by comparing the retention time and UV spectrum of the sample using the standard sample with a known concentration (Supplementary Table S1), and the content of phenolic compounds was expressed based on fresh weight as μg kg^−1^.

### 4.6. Analysis of Lycopene and Ascorbic Acid

Lycopene was extracted according to the method of Serino et al. [61]. Concretely, 0.5 g frozen powder sample was fully mixed with 0.1 mL NaCl saturated aqueous solution and 0.05 mL *n*-hexane in a vortex mixer for 30 s, followed by a centrifugation at 13,200 5× *g* for 2 min at 4 °C. Afterwards, 0.2 mL dichloromethane and 1 mL ethyl acetate were added into the mixture, repeating the steps above once, then the supernatant was collected after centrifugation. The sample was filtered through a 0.45-μm membrane filter before the HPLC assay. Lycopene content was measured referring to Bu et al. [62] by HPLC, using a Zorbax SB-C18 column (silica 5 μm, 4.6 mm × 250 mm, Agilent, USA) and a Shimadzo LC2012A pump (Shimadzo Corp., Tokyo, Japan) under 475-nm detection wavelength. Lycopene concentration was quantified by a calibration curve prepared as standard, and the result was expressed based on fresh weight as mg 100 g^−1^.

Ascorbic acid was determined as described by Jagadeesh et al. [63]. About 5 g of frozen-powder sample was homogenized with 20 mL of a buffer solution, containing 1 g L^−1^ oxalic acid and 4 g L^−1^ anhydrous sodium acetate. After extraction for 10 min at 4 °C, the homogenate was centrifuged at 9000× *g* for 15 min at 4 °C and the supernatant was collected. 10 mL of supernatant was used to titrate against a solution consisting of 295 mg L^−1^ DPIP and 100 mg L^−1^ sodium bicarbonate. A standard solution of ascorbic acid comprising various concentrations (0.1 g L^−1^, 0.01 g L^−1^, 0.005 g L^−1^, 0.0025 g L^−1^, 0.00125 g L^−1^) was used to quantify ascorbic acid in the samples. The result was expressed based on ascorbic acid and fresh weight as mg 100 g^−1^.

### 4.7. Analysis of Enzymatic Activities

About 1.0 g of frozen powder was homogenized in 4 mL of 0.1 M cold sodium borate buffer (pH 8.8, 1.0 mM EDTA, and 3% polyvinylpyrrolidone) for the phenylalanine ammonia-lyase (PAL) assay or 0.05 M cold sodium phosphate buffer (pH 7.8, 1 mM EDTA, and 2% polyvinylpyrrolidone) for peroxidase (POD), polyphenol oxidase (PPO), catalase (CAT), and ascorbate peroxidase (APX) assays, respectively. The mixture was centrifuged at 9000× *g* for 15 min at 4 °C, then the supernatant was used for the analysis of enzymatic activities.

PAL activity was determined based on the method of Yingsanga et al. [64] with some modifications. A total of 4 mL of reaction mixture consisted of 2 mL of 0.1 M sodium borate buffer (pH 8.8), 1 mL of 20 mM l-phenylalanine, and 1 mL of supernatant. The mixture was incubated for 1.5 h at 37 °C. One unit of PAL was defined as the amount of enzyme that caused an increase of 0.1 in absorbance per hour at 290 nm.

POD activity was measured with guaiacol as described by Zhang and Kirkham [65]. 0.1 mL supernatant and 20 μL of 40 mM H_2_O_2_ were mixed with 2.83 mL of 10 mM sodium phosphate buffer (pH 7.0) and 50 μL of 20 mM guaiacol. One unit of POD activity was defined as a change of 0.1 per min at 470 nm due to guaiacol oxidation.

PPO activity was assayed according to Zhang and Kirkham [65] with slight modifications. 0.1 mL supernatant was added into 2 mL of 50 mM sodium phosphate buffer (pH 6.0) and 1 mL of 50 mM pyrocatechol. One unit of PPO activity was defined by following the change of 0.1 in absorbance at 425 nm per min.

CAT activity was conducted using the method of Nahakpam and Shah [66] with some modifications. The reaction mixture comprised 1.5 mL of 50 mM sodium phosphate buffer (pH 7.0) and 0.2 mL supernatant; the reaction was started by adding 0.3 mL of 10 mM H_2_O_2_. One unit of CAT activity was defined as a decrease of 0.1 in absorbance at 240 nm per min.

APX activity was performed according to the method described by Nahakpam and Shah [66] with some modifications. The reaction mixture contained 2.6 mL of 50 mM sodium phosphate buffer (pH 7.0), consisting of 0.1 mM EDTA and 0.5 mM ascorbic acid, and 0.1 mL supernatant. Afterwards, 0.3 mL of 2 mM H_2_O_2_ was added to initiate the reaction. One unit of APX activity was defined as the change of 0.1 in absorbance at 290 nm per min.

The results of enzymatic activities were expressed based on fresh weight as U g^−1^.

### 4.8. Analysis of Antioxidant Capacities

The antioxidant capacities were determined as ferric reducing antioxidant potential (FRAP) and 1,1-diphenyl-2-picrylhydrazyl (DPPH) radical scavenging activity. The extraction method of FRAP and DPPH assays was mentioned in Section 2.4 above. FRAP was conducted as described by Alothman et al. [67] with little modification. Briefly, 0.1 mL of extract was mingled with 3.6 mL of FRAP reagent and the mixture was incubated at 37 °C for 0.5 h. The absorbance of the sample at 593 nm was determined using a spectrophotometer, and the result was expressed as μmol g^−1^ Trolox. DPPH radical scavenging activity was measured according to the previous method [68]. In general, 0.2 mL of extract was mixed with 2.8 mL of 60 μM ethanolic DPPH. The reaction mixture was kept in the dark for 0.5 h at room temperature. Afterwards, the absorption of the sample was measured at 515 nm and the result was expressed as the percentage of DPPH radical inhibition (DPPH%).

### 4.9. Analysis of Gene Expression

Genes related to phenolic compounds were analyzed by quantitative real-time PCR (qRT-PCR) and the sequences of primers designed with Primer 5.0 software were shown in Supplementary Table S2. The gene expression was analyzed according to the method of Bu et al. [69]. Generally, total RNA from eight random fruit samples were isolated with RNAiso Plus (TaKaRa, Shiga, Japan) according to the manufacturer’s instructions. Afterwards, RNA concentration was quantified by a Nanodrop spectrophotometer (NanoDrop 2000, Thermo-Fisher Scientific Inc., Bartlesville, OK, USA). The isolated RNA was reverse-transcribed to single-strand cDNA using a PrimeScript RT kit (TaKaRa, Shiga, Japan). qRT-PCR was performed using a SYBR^®^ Premix Ex TaqTM kit (TaKaRa, Dalian, China) according to the manufacturer’s protocol on an ABI Step One RT-PCR System (Applied Biosystems, Beverly, MA, USA). The relative quantity of gene expression was analyzed using the comparative Ct method and calculated with 2^−ΔΔCt^ method [70]. Each measurement was performed with three replicates.

### 4.10. Statistical Analysis

All experiments were performed using a completely randomized design, and were repeated three times. All values were expressed as mean ± standard deviation. Data were analyzed by SPSS Statistics Version 20.0 (Cary, NC, USA). Differences at *p* < 0.05 were considered significant when the means were compared by Student’s *t*-test. Correlation analysis between gene expressions, phenolic compounds, and antioxidant capacities was executed with Pearson’s correlations and two-sided tests based on the relative values (RVs) of all indexes in ABA-treated fruits on days 1, 7, 11, and 15.

## 5. Conclusions

The results of the present study indicated that exogenous-ABA treatment induced the expressions of *PAL*1, *C4H*, *4CL2*, *CHS2*, *F3H*, and *FLS*; promoted PAL, POD, and PPO activities; accelerated the accumulation of total phenolics, total flavonoids, and phenolic compounds; and enhanced lycopene and ascorbic acid contents, as well as the antioxidant enzymatic activities (CAT and APX) and antioxidant capacities (FRAP and DPPH) during tomato ripening. These results suggest that ABA plays a vital role in activating the phenylpropanoid pathway and improving bioactive components and antioxidant capacities of the postharvest tomato during ripening. Further studies are required to explore in more depth the molecular mechanisms triggered by the ABA treatment during tomato ripening.

## Supplementary Materials

The following are available online at https://www.mdpi.com/1420-3049/25/6/1346/s1, Table S1: The concentrations of the standard phenolic compounds selected in this research. Table S2: Sequence of primers used for qRT-PCR analysis.
