# Supplementary material for: Effects of Exogenous Abscisic Acid on Bioactive Components and Antioxidant Capacity of Postharvest Tomato during Ripening
Source: Molecules. 2020 Mar 16;25(6):1346. doi: 10.3390/molecules25061346 (PMC7144105; doi:10.3390/molecules25061346)
Supplement: Supplementary file 1 [file molecules-25-01346-s001.pdf]

Supplementary

Effects of Exogenous Abscisic Acid on Bioactive Components and Antioxidant Capacity of Postharvest Tomato During Ripening

Xiaoya Tao [1,†], Qiong Wu [2,†,*], Halah Aalim [1], Li Li [1], Linchun Mao [1], Zisheng Luo [1] and Tiejin Ying [1,*]

[1] College of Biosystems Engineering and Food Science, Fuli Institute of Food Science, Zhejiang Key Laboratory for Agro-Food Processing, Zhejiang R & D Center for Food Technology and Equipment, Zhejiang University, 310058 Hangzhou, China; taoxiaoya00@163.com (X.T.); halahaalim@zju.edu.cn (H.A.); lili1984@zju.edu.cn (L.L.); linchun@zju.edu.cn (L.M.); luozisheng@zju.edu.cn (Z.L.)

[2] Collaborative Innovation Center of Henan Grain Crops, Henan Collaborative Innovation Center of Grain Storage and Security, School of Food Science and Technology, Henan University of Technology, 450001 Zhengzhou, China

* Correspondence: qiongwu0605@126.com (Q.W.); tjying@zju.edu.cn (T.Y.); Tel.: +86-371-67758022 (Q.W.); Tel.: +86-571-88982174 (T.Y.)

† These authors contributed equally to this work.

Table S1. The concentrations of the standard phenolic compounds selected in this research.

Phenolic compound name	Retention time (min)	Concentration (μg mL^{-1} methanol)
gallic acid	5.139	100
3,4-dihydroxybenzoic acid	8.683	290
(+)-catechin	13.821	100
p-hydroxybenzoic acid	18.685	290
chlorogenic acid	23.137	50
vanillic acid	26.762	230
caffeic acid	29.28	200
p-coumaric acid	35.783	200
syringic acid	37.791	170
ferulic acid	39.431	200
sinapic acid	49.719	240
isoquercitrin	52.306	200
ellagic acid	52.77	180
cinnamic acid	59.454	160

rutin	60.016	300
quercetin	61.026	120

Table S2. Sequences of primers used for qRT-PCR analysis.

Gene name	Forward primer (5′-3′)	Reverse primer (5′-3′)
PAL1	ACTTGAGGCATTTGGAAGA	CATCAGTGGGTAGTTAGCG
C4H	CAGGGAAGGGTCAAGATA	CAATCCCATTTGTAGCAG
4CL2	GCTCTGCCGTATTCCTCC	CCGTCATTCCATAACCCT
CHS2	GCCGACTACCAACTCACC	AGGGCTTGTCCAACCATAC
CHI	AATGAAGTGATGGTGGATG	AAACTTGTCAACTGGAGCA
F3H	ATCTGGTGCTGGATTCTGG	ACTCTTGGGCTCAAACGAC
FLS	ACAGGGAAGCAAATGAGGA	TTGGGACAAGAAGGGTGAT
Actin	CACCATTGGGTCTGAGCGA	GGGCGACAACCTTGATCTT
